# Anomaly Detection in Traffic Surveillance Videos Using Deep Learning

**DOI:** 10.3390/s22176563

**Published:** 2022-08-31

**Authors:** Sardar Waqar Khan, Qasim Hafeez, Muhammad Irfan Khalid, Roobaea Alroobaea, Saddam Hussain, Jawaid Iqbal, Jasem Almotiri, Syed Sajid Ullah

**Affiliations:** 1 Department of Information Technology, University of Sialkot, Sialkot 51040, Pakistan; 2 School of Physic Engineering and Computer Science, University of Hertfordshire, Hatfield AL10 9AB, UK; 3 Department of Information and Electrical Engineering and Applied Mathematics, University of Salerno, 84084 Fisciano, SA, Italy; 4 Department of Computer Science, College of Computers and Information Technology, Taif University, P.O. Box 11099, Taif 21944, Saudi Arabia; 5 Department of Computer Science, Capital University of Science and Technology, Islamabad 44000, Pakistan; 6 School of Digital Science, Universiti Brunei Darussalam, Jalan Tungku Link, Gadong BE1410, Brunei; 7 Department of Information and Communication Technology, University of Agder (UiA), N-4898 Grimstad, Norway; 8 Department of Electrical and Computer Engineering, Villanova University, Villanova, PA 19085, USA

**Keywords:** deep learning, video classification, accident detection, surveillance system, anomaly detection

## Abstract

In the recent past, a huge number of cameras have been placed in a variety of public and private areas for the purposes of surveillance, the monitoring of abnormal human actions, and traffic surveillance. The detection and recognition of abnormal activity in a real-world environment is a big challenge, as there can be many types of alarming and abnormal activities, such as theft, violence, and accidents. This research deals with accidents in traffic videos. In the modern world, video traffic surveillance cameras (VTSS) are used for traffic surveillance and monitoring. As the population is increasing drastically, the likelihood of accidents is also increasing. The VTSS is used to detect abnormal events or incidents regarding traffic on different roads and highways, such as traffic jams, traffic congestion, and vehicle accidents. Mostly in accidents, people are helpless and some die due to the unavailability of emergency treatment on long highways and those places that are far from cities. This research proposes a methodology for detecting accidents automatically through surveillance videos. A review of the literature suggests that convolutional neural networks (CNNs), which are a specialized deep learning approach pioneered to work with grid-like data, are effective in image and video analysis. This research uses CNNs to find anomalies (accidents) from videos captured by the VTSS and implement a rolling prediction algorithm to achieve high accuracy. In the training of the CNN model, a vehicle accident image dataset (VAID), composed of images with anomalies, was constructed and used. For testing the proposed methodology, the trained CNN model was checked on multiple videos, and the results were collected and analyzed. The results of this research show the successful detection of traffic accident events with an accuracy of 82% in the traffic surveillance system videos.

## 1. Introduction

The process by which behavior and activities are monitored for managing and protecting people is called surveillance. The most popular method for observing interesting objects from a distance is using Internet of Things (IoT) devices such as closed-circuit television (CCTV) cameras. An improvement in quality of life is achieved by implementing artificial intelligence in IoT devices [1,2]. In the past few years, vast numbers of CCTV cameras were installed in various public and private areas for security and monitoring anomalous activities and traffic. These cameras can help in finding anomalies and can take action accordingly [3]. Currently, anomaly detection is an emerging topic for researchers in different domains such as industry, business, mobile fraud, health, and people’s local activities [4,5,6]. Anomaly detection is very beneficial to protect and save the properties of public and personal assets. Surveillance cameras are used for security purposes or to find anomalous events [7] or irregularities [8] in public or private places. In 2017, 954,261 CCTV cameras were installed in public places in South Korea, which was a 12.9% increase from the previous year [9]. Surveillance cameras are providing the facility to detect anomalous activities and abnormal behavior in the real world.

Image-based anomaly detection (AD) is the process of identifying an image that is not visible as being an image of the regular classes (inliers) or as the anomalous classes (outliers) by learning from normal images used for training. This is of great significance in real-world situations, such as the monitoring of anomalous events from cameras for surveillance, since the majority of data are normal, but the detection of abnormal data is necessary. One major challenge in image anomaly (AD) detection is learning discriminative data solely from normal-looking images in class training [10].

The transportation sector also faces many issues with the increase in population, such as traffic congestion, traffic jams, and traffic accidents. In the modern world, new technologies are used to overcome these kinds of traffic issues. In almost all developed countries, video-based camera surveillance systems (VCSSs) are implemented across local roads, railway stations, airports, streets, highways, car parks, motorways, and other high-priority locations [11]. VCSSs decrease the human involvement in monitoring and capturing traffic activity, automatically present the user with a real-time feed of important remote locations and store the feed in the form of videos. Installed VCSSs are being used for detecting and tracking vehicles, the classification of vehicles, the estimation of the flow of traffic, abnormalities, vehicle accidents, etc., and are actively being used by law enforcement authorities and other departments.

The installation of a VCSS does not eliminate human intervention completely, as a person is required to observe the output at any given time to tackle any anomaly such as a traffic accident, blockage, etc. Researchers in this field are also trying to automate this part by coming up with sophisticated algorithms and solutions for anomaly detection from videos. Recent research in anomaly detection proved that binary classification (normal and abnormal) provides accurate and effective results [12]. However, this method is limited because the videos of anomalous events are hard to collect due to their scarcity. Therefore, researchers have also presented a new category of techniques that require low to no supervision, including dictionary learning [13], spatiotemporal features [14,15], and autoencoders [16], which train the computation models on normal activities and the normal behavior of the monitored environment. Finding a dataset for the normal behavior of the environment is not difficult, as most of the available surveillance data are comprised of normal activities. During the testing phase, these techniques were able to detect anomalous events due to deviations from normal activities.

This research is focused on detecting a type of anomaly that is a traffic accident from videos using a convolutional neural network (CNN), which is a special type of artificial neural network. For traffic surveillance, a VCSS with some additional capabilities is used, which is called a video-based traffic surveillance system (VTSS). To train the CNN model to detect anomalies in a video, a large amount of data are required, as the model is trained to recognize different types of traffic accidents. The VTSS enables the production of the data that are needed for training the CNN model. With the passage of time, the VTSSs are being enhanced and optimized in collecting, storing, and cleaning data, so there is a decent amount of content available online. Nevertheless, the continuous growth of data and the addition of different types of anomalies requires the proposed methodology to be generalizable, which makes the detection of anomalies a challenging task.

In this research, the traffic accident events are targeted to be detected by analyzing frame images in VTSS videos to enable helping those people who are helpless during the accident. Moreover, the system helps the staff that monitors the multiple screens of the VTSS. This research uses a supervised algorithm, i.e., a CNN to learn the distinct features of anomalous behavior, which is performed by training the CNN model using images showing the traffic accident. The convolutional autoencoder stack in the CNN model is used to learn the spatial patterns from images. The trained CNN model is then used to classify traffic video frames for which results are collected and analyzed as part of the experimentation of the proposed methodology.

VTSS is a traffic surveillance system that is used for monitoring traffic. The video feed provided by the VTSS is then used to detect any abnormal activity and take action accordingly. Human involvement is mandatory to observe the anomaly from the VTSS. This research is targeted to devise a technique to automatically detect traffic accidents, which are a type of anomaly, in a video.

### Contribution

- In this research we propose an enhancement in the VTSS by integration with our proposed technique to automatically detect accidents in a video feed.
- We propose a novel method of classifying video by integrating the output of the CNN model rolling average prediction algorithm.
- While performing this research, a vehicle accident image dataset (VAID) was created for training the CNN. The dataset consists of 1360 images captured by normal cameras and the VTSS.

The overall structure of the article is summarized below: Section 2 examines related works, while Section 3 briefly defines the proposed methodology. Section 4 is about the implementation. Section 5 is related to the experiments and elaborates on the results and discussion, and Section 6 provides the conclusion.

## 2. Related Works

In related works, we studied surveillance systems and how they work. We also studied how it is helpful to find anomalous activity in public or private places and highways or motorways. This also helped us to find which deep learning algorithm is suitable for image or video analysis.

The process of monitoring behavior and activity to manage and protect people is called surveillance. The surveillance system is mostly used by medical and security organizations. The best method of surveillance is to observe objects and people from a long distance with help of cameras. These are called CCTV cameras, and this system’s name is the video camera surveillance system (VCSS). The video surveillance system has been very popular for the past few years due to its low hardware cost and high security concerns. These systems are used to detect anomalous behavior and identify irregular activity and abnormal patterns in video data [17,18]. 

In the modern era, these surveillance systems are also used to follow or monitor traffic. These systems are traffic surveillance systems (VTSS). These systems create a large amount of data in the form of videos. Varun Chandola [7] explained anomalies and their types. An anomaly is a form of patterns in data that do not behave normally. These patterns are mentioned as anomalies, surprises, discordant, expectations, outliers, peculiarities, observations, or contaminants in various application domains. Anomaly detection is a method that is used to find those irregular patterns from the data that follow the expected behavior. Many authors or researchers carried out their research on anomaly detection based on image and video classification. Mehrsan Javan and Martin D. Levine [19] described the method of finding anomalies in dense videos based on spatiotemporal volume. The training provides examples of normal behaviors. This kind of event occurs in real-time from densely sampled video when captured in the spatiotemporal region of anomalous activity. By using a probability framework, the video volume is modeled, which is based on the spatiotemporal composition. The framework calculates the likelihood of normal behavior in the video. The probability framework is used. It decreases the time to compute similarity. This technique is much quicker than the IBC and MDT methods. M. Sabokrou, M. Fathy, and M. Hoseini [20] presented an accurate and fast method for the localization of anomalies and anomaly detection in video. They used a cascade classifier for anomaly detection in the video, in which two phases were involved based on the analysis of SV (spatial value) and RE. This method was proposed for fast anomaly detection.

Xinyi Cui et al. [21] presented a new technique to describe the current behavior condition of a subject based on the position/velocity of the subject and its neighbors. They used the function of interaction energy potential. For this technique, the social behavior was captured by the relationship between its action and energy potential. For this technique, they used a support vector machine to consider the rare energy–action pattern as an anomaly. This proposed method takes advantage of the relation between a current person’s condition and their reactions. Moreover, this technique is not dependent on human tracking and detecting algorithms, and its performance is much better on both the BEHAVE and UMN datasets.

Cem Direkoglu and Melike Sah [22] presented a novel feature-based optical flow used for the detection of abnormal behavior of a crowd. It works on the pixel level. This method takes angle differences at each pixel level to find anomalous behavior. Moreover, it compares the angle difference of the current frame with the previous one. To detect normal behavior, a simple one-class support vector machine is used. Moreover, they performed experiments on the UMN and PETS2009 datasets. Shifeng Li and Chunxiao Liu [23] introduced the MAP framework for anomaly detection using prior knowledge. The Bayesian framework is integrated with prior knowledge for detecting an anomaly. The likelihood function is computed by using the maximum grid template. This experiment provides very effective results in complex situations.

Habib Ullah [24] described the flow of pedestrians and found anomalies in a crowded area. He proposed the (GKIM) model for the detection of anomalous objects and localization in pedestrian flow. He calculated the EER and DR on both the pixel level and frame level. In the frame-level analysis, the anomalous entity detection is based on its location, and in the pixel-level analysis this method localizes the anomalous entity in terms of its pixel.

In a method by V. Ravindran [25], image handling and ML were used to automatically detect road accidents using the camera static image, which was placed on roads and streets. A single frame is the combination of three steps that allow you to analyze accidents using the static picture. This uses SVMs that were trained on HOG geographics and geographical GLCM. This stage proposes a method to automatically find road accidents. The second step is an overseen learning technique that detects scratched cars as a standing image series. This is a new category of objects that machine vision methods have not been able to see. The final stage includes two datasets of damaged car DCD-1 and DCD-2, which are free. This modification is based on the distance to capture the quality of the images. The system’s accuracy is 82 % in damaged cars dataset-1, which was caught at approximately 2 m using high excellence. The accuracy was 64% in DCD2, which captured the next 20 m with the same feature. The machine vision technique was successful in locating broken cars. This project has one major drawback: it cannot find damaged cars that have been severely damaged.

Shivangi [26] proposed a model based on IoT to identify accidents and inform the authorities as soon as possible. The car would be equipped with intelligent sensors and microcontrollers that could trigger during an accident. You can combine other modules with this system to reach an accident site and then send them to a nearby hospital to report the event. This wireless communication mode sends messages using GPS and GSM.

Ahmar Azam [27] proposed an IoT-based accident prevention system using a detector for checking alcohol and an accelerometer. Arduino Nano is the alcohol sensor. A GSMC modem, or GSM, handles the SMS. The vehicle’s motor is shown using GPS (global positioning system), an LCD display, and a CPU fan. The device used for reading alcohol detects the output of equivalent resistive, which supports alcohol absorption. The GSM module allows transmission between mobile phones, the accident detection system, and the notifying system. GPS locates the accident location and sends these microwave signals toward satellites.

G. Liang [28] presented the automatic detection of accidents with the help of an SVM modified by the algorithm of ant colony (ACA). This allows for traffic accidents to be predicted. The Internet of Things (IoT), which is the overall mechanism of intelligent transportation, is used here. They used technology such as RFID as well as wireless communication. These are used for real-life movement records for prediction and standard road traffic setups to improve the assessment of the situation. The SVM reformed and improved by ACA was able to achieve faster merging speeds. Moreover, the mean square errors, which are related to simple SVMs, were more severe. The ant colony algorithm proved to be efficient.

Tian wang [29] proposed a framework for analysis and representation. Without object tracking, it extracts visual features based on optical flow. It uses one-class SVM and PCA to find abnormal behavior. It is a supervised learning technique, as they want it to learn the regular behaviors. This process has main three steps. First, using the HornCSchunk (HS) optical flow technique in greyscale, it calculates the optical flow of individual frames. Second, each frame calculates the HOFO (histogram of optical flow orientation). To save time, it does not consider the background area. Therefore, it uses the above two classifiers for the classification of surveillance video features. It shows good results on the PET scene and the UMN dataset. The limitations of this algorithm are there is no training online and there are high false alarm rates. Due to the extensive availability of data and processors at cheaper rates, in many domains deep learning approaches proved their remarkable performance in areas such as anomaly detection [30], object detection [31,32], topic modeling [33], image classification [34], semantic segmentation [35], visual tracking [36,37], representation learning [38], etc.

Ren et al. [39] expressed the spatiotemporal correlation effect in the data related to traffic accidents and created a model of a recurrent neural network for the prediction of accident risk. It is a proactive system to appraise related traffic accidents. The extra features such as traffic and the characteristics of the road can also be used to improve the accuracy. Bortnikov et al. [40] created the accident detection model, a 3D convolutional neural network. For training the model, they produced the traffic videos in games. Two loss functions were applied for testing with and without optical flow. This model was applied to real-world traffic videos. Tian et al. [41] proposed the model of deep learning using the object detection algorithm you only look once (YOLO) for car accidents. It was also used to gather other information such as accident location. To obtain accuracy and real-time detection, this model has a multi-scale loss function and technique for feature fusion with vital weights.

Ohgushi et al. [42] used an autoencoder with segmentation to detect obstacles on roads. The encoder in autoencoder is a semantic image generator, and the decoder is a photographic image generator. This technique works in an unsupervised way such that the model is trained on a normal road. Aside from another deep learning approach to traffic surveillance presented by Yao et al. [43], the unusual activity and object tracking techniques are used. The unsupervised learning approach is used for videos captured by dashboard cameras. In this technique, the model is trained on normal events. During testing, the patterns that deviate from normal are considered anomalies.

Zhang Minli [44] described an artificial neuron network. An ANN is highly inspired by the biological nervous system (such as the human brain). The ANN consists of three layers: the input, output, and hidden layers. The hidden layer can consist of one or more layers. Each layer has different numbers of neurons. Their transmission is forward or reversible. The input x is given by the input layer. Some processes are performed on this input by the hidden layers, and y is the predicted value that is the result generated by the output layer. ANNs have many advantages but the major advantage is that they can easily solve uncertainty, multiple factor, and non-linear problems.

Keiron O’Shea [45] briefly described convolutional neuron networks (CNNs). They are one form of ANN. They are basically used to recognize patterns from images. Moreover, they are used to decrease the parameter for building the new model. CNNs have five multiple layers: the input, convolutional, pooling, fully connected, and output layers. The input layer has the values of the pixels in the image. The convolution layers apply some weight of the values and obtain the scalar product of both. The ReLu is used to apply activation functions such as sigmoid on the output of the previous layer. The pooling layer is used to decrease the spatial dimensionality of any input and decrease the number of parameters on that activation. Fully connected layers work like simple ANNs. They obtain a score from the activation function and use it for classification. ReLu can be used in these layers to increase performance.

Andrej karpathy and George Toderici [46] briefly discussed the performance of CNNs in a large number of video classifications. They obtained CNN architectures with the ability to learn features from partially labeled data. They used the UCF-101 dataset for the experiment. It was observed that the single-frame model always shows better performance. They also said that a mixture architecture that consists of the stream of high-level resolution and low-level context will be an effective way to increase the speed of CNNs without losing accuracy. Moreover, when they retrained three layers of the CNN, they obtained the highest transfer learning rate.

Guilhem Cheron and Ivan Laptev [47] proposed a new approach to the convolution neural network named is pose-based convolution neural network (P-CNN). They aimed to recognize human action in video. By tracking human body parts, they collected information about their motion. They also implemented this proposed approach on the JHMDB and MPII cooking datasets. For both datasets, the proposed approach showed improvement over the state-of-the-art approaches.

CNN also proved itself by correctly predicting the anomalous pattern of disease from medical images such as brain tumor from MRI images [48], coronavirus disease from chest X-rays [49], and early breast cancer detection [50]. An automated strategy is used for abnormality classification as well as localization and report retrieval for abnormalities that have been identified. MSDNet presents an ensemble of convolutional neural models to classify abnormalities, which blends the capabilities of several CNN models to improve the accuracy of abnormality classification. The proposed model was also designed to locate and show the anomaly that was detected on the radiograph, using an abnormal region detection algorithm that further improves the diagnostic quality.

In contrast to traditional methods, the CNN can detect the characteristics of vibration time domain signals automatically without processing. To achieve an intelligent diagnosis and enhance the accuracy of recognition, the WKCNN model follows the idea of widening convolution kernels so as to achieve a greater receptive area and proposes a design pattern that is based on this concept [51].

The anomaly detection process of the intelligent monitoring network of expressways, which is based on edge computing and deep learning, is being investigated to improve real-time expressway monitoring. The edge processing server sends video data from the cameras that are part of the intelligent monitoring structure to be screened and then transferred to the convolutional networks. The CNN model employs an optical flow histogram, which is the multi-scale technique to process the video footage following the edge calculation to produce the sample sets for training, and forwards these data to the AlexNet model to be used for feature extraction [52].

Other machine learning algorithms such as RNN and LSTM are mostly used for data processing and language processing. The above literature review proves CNN does more to help us to perform visual analysis (images and video). The comparison of the related works is shown in Table 1.

## 3. Methodology

**Figure 1:**
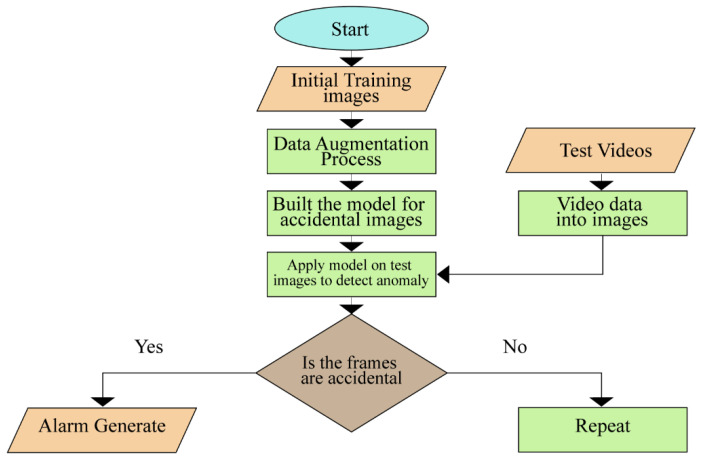
Flowchart of methodology. The first step is to initialize the training images. The second step is a data augmentation process. The third step is to build the model for accident images. The fourth step is applying the model to images. The fifth step is the detection of an anomaly in frames. The final step is to generate the alarm or repeat the algorithm.

The proposed approach begins with gathering accident or car crash images using videos from VTSS. The machine learning (ML) model under the family of artificial neural networks known as convolutional neural networks (CNN) was used to create an anomaly detection model. The anomaly detection model was then trained on a subset of the collected data. In the end, the trained model was used to classify the unlabeled data. The results were then compared to the actual outcomes in order to perceive the performance of the model in anomaly detection. The overall flowchart of the methodology is shown in Figure 1.

### 3.1. Convolutional Neural Network in Deep Learning for Anomaly Detection

**Figure 2:**
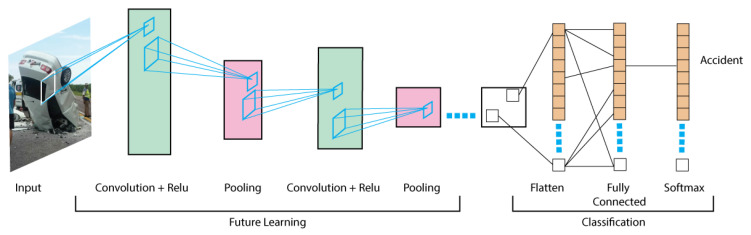
Architecture of CNN for anomaly detection. In this architecture, after providing input images, two main processes are implement on them, future learning and classification. The future learning process consists of convolutional layers with activation functions and pooling layers. The classification process consists of a flattening layer, a fully connected layer, and a softmax activation function.

Deep learning (DL) is a category of artificial neural networks (ANNs). The ANNs in DL consist of at least three layers, where the first layer is for input, the last layer is for output, and the layers in between are hidden layers [53]. The overall architectural of a CNN for detecting an anomaly is shown in Figure 2.

CNN is one of the leading categories of neural networks for image classification and image recognition, as observed in a review of the literature [54]. A typical CNN model consists of five layers:

- Input layer;
- Convolution layer;
- Pooling layer;
- Fully connected layer;
- Output layer.

**Figure 3:**
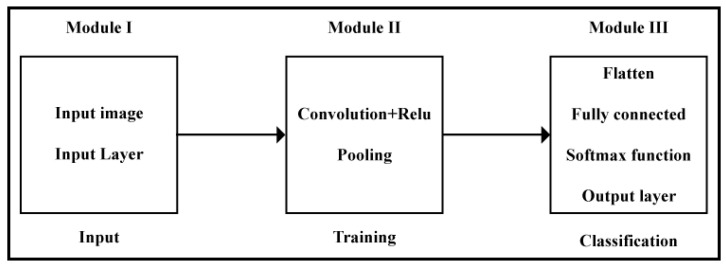
Block diagram of CNN modules. It consists of three main modules. The first module is input. It consists of an input layer or input image. The second module is training. It consists of convolutional and pooling layers. The third module is classification. It consists of a fully connected layer, an activation function, and an output layer.

The overall architecture of a CNN is based on three modules. The block diagram of the modules of CNN is in given in Figure 3, including:

- Input;
- Training;
- Classification.

#### 3.1.1. The Numbers of Parameters in CNN

In the case of a CNN, every layer is comprised of two types of parameters: weights and biases. In total, the number of parameters used is the total of all the biases and weights.

#### 3.1.2. Input Image

**Figure 4:**
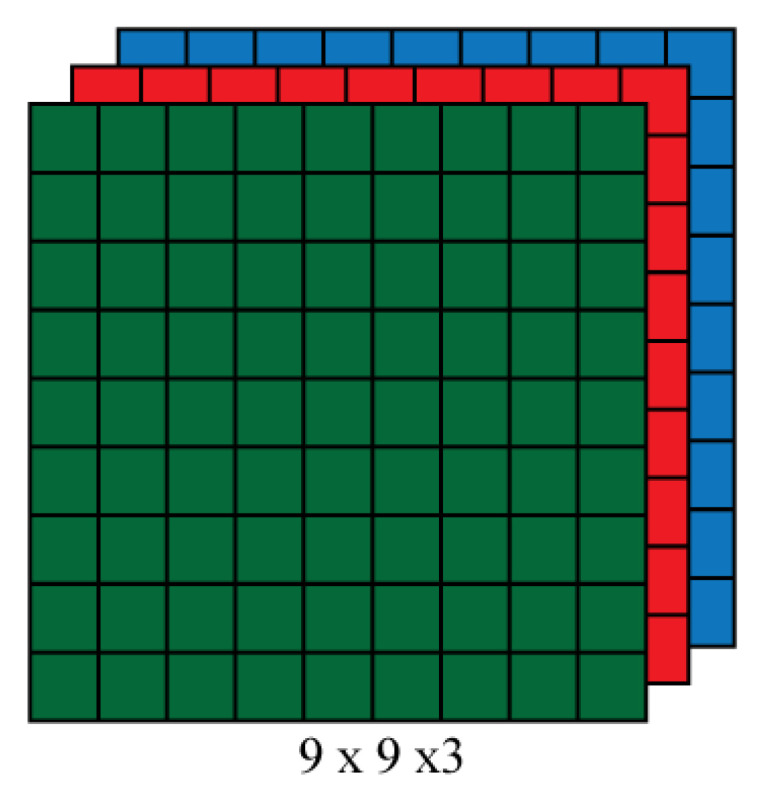
Array of an RGB matrix. Here, it is represented by nine columns, nine rows, and three channels.

An image consists of matrices of pixels in digital form, as used and stored by a computerized system. The number of these matrices depends upon the type of picture, and the dimensions of these metrics depends on the resolution of the image. Figure 4 describes how an image dimension can be represented as h × w × d, where h is the height of the image, w is the width, and d is the number of matrices representing an image. The value of d is 3 in the case of an RGB image and 1 for a grayscale image.

#### 3.1.3. Input Layer

The input layer of a CNN model is responsible for transferring the image data into the network. In a CNN model, the input layer consists of a matrix that accepts the input image as a parameter and forwards the image data to the next layers. If the image consists of multiple matrices, then the image is converted to a single matrix before providing it to the input layer using other available techniques.

#### 3.1.4. Convolutional Layer

The second layer of the CNN model is the convolutional layer, which extracts the features from the given image as inputs. To extract features from the input, this layer uses a small square of input data that is equal to the size of the kernel or filter matrix. The kernel matrix is a user-defined matrix used to keep the specific parts of the image matrix expressed in Equations (1) and (2), where h is the height of the image, w is the width of the image, d represents the number of channels in RGB, and f is the filter.

Image matrix (h × w × d) Filter (fh × fw × d)(1)

Output (h − fh − 1) × (w − fw − 1) × 1(2)

After a pass of the kernel matrix on the whole image, a new matrix that is reduced in size and noise and only captures the point of interest from the image is created, which is called a feature map. The convolutional layer also contains the ReLU (rectifier linear unit) as an activation function, which allows the convolution layer to keep only the positive data and to add non-linearity to the CNN model. The ReLU function is given in Equation (3).

f (x) = max (0, x)(3)

#### 3.1.5. Pooling Layer

The job of the pooling layer is to minimize the spatial mass of the image after a convolutional layer. The pooling layer is mostly used to reduce the size of the input image while keeping the important features of the image intact. Another reason for adding the pooling layer is to cut the cost of the computation, as after this layer the image data are provided to a neural network. In the pooling layer, a filter is computed over the output of the convolutional layer. There are two parameters for the pooling layer, one is a filter, which is a small matrix, and the other is a stride, which denotes the number of steps to move a filter after computation. Every computation of the filter over the data results in a single value that is used to create a small matrix for the input image while keeping the significant information. The dimension of the output matrix of the pooling layer depends on the size of the filter, stride, and input image. Multiple types of pooling are used in practice:

- Sum pooling;
- Average Pooling;
- Max Pooling.

#### 3.1.6. Flattening

**Figure 5:**
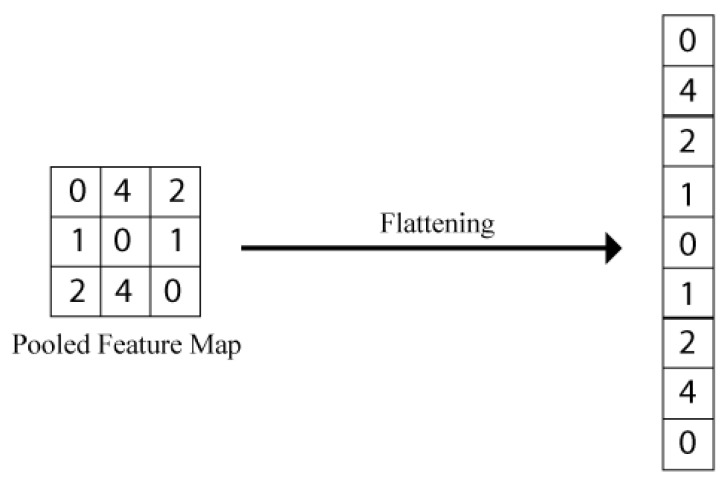
The flattening technique presents the whole matrix in one column.

The matrix output by the pooling layer is first subjected to a flattening layer before forwarding the information to the neural network. The job of the flattening layer is to convert the matrix into an array of values with a width of 1. The purpose of this transformation is to make the input matrix compatible with the neural network, as the neural network is not able to process the matrix-like data and is only able to process a feature vector. A visual representation of this transformation is given in Figure 5.

#### 3.1.7. Fully Connected Layer

The next layer in the CNN model is the fully connected layer. These layers can be one or multiple and are comprised of neurons. The neurons in these layers are connected to every neuron in the next layer. These layers act similarly to the hidden layers in the neural network, where the same learning process takes place to learn the features extracted from the image by the previous layers. The purpose of this layer is to learn the features of the images and classify the preceding images based on the learned characteristics.

#### 3.1.8. Softmax Function

Softmax is an activation function used in the output layer of a CNN. The fully connected layer can use any type of activation function, but the reason for using Softmax in the output layer of a CNN is that it provides the probability distribution over the classes that are used to assign a class to the input images. The output of the Softmax function is a vector that provides the list of possible outcomes that represent the probability distribution. The Softmax function is given as follows in Equation (4). In this equation, *S* is Softmax, *y_i_* represents the input vector, and a standard exponential function of the input function is represented by *e^y_i_^*.

(4) $$S\left(y_{i}\right)=\frac{e^{y_{i}}}{\sum_{j}\;e^{y_{i}}}$$

#### 3.1.9. Output Layer 

The last layer of the fully connected layers is the output layer for a CNN, and it is described above. This layer uses the Softmax function as the activation function, which outputs a value between 0 and 1, showing the likelihood of an image belonging to a specific class. The number of neurons in the output layer is equal to the number of classes in which an image needs to be classified. All neurons in the output layer show the probability distribution of the image for the classes, and the class with the highest probability is assigned to the image.

### 3.2. Keras

This research uses the Keras library for the implementation of the CNN model. Keras is an effective and easy-to-use open-source Python library for developing artificial neural networks [55]. Keras provides a wrapper around well-organized numerical computational libraries such as tensor flow and Theano and provides comprehensive APIs around them, which allows users to train and developed neural network models in a few lines of code.

### 3.3. Previous Datasets

To find a dataset of traffic accidents, a detailed examination of commonly used video anomaly detection datasets was conducted, which included BOSS [56], UMN [20], avenue [14], subway entrance [57], subway exit [57], UCSD Ped1 [58], UCSO Ped2 [58] and UCF [59]. The UCF dataset [59] consists of long surveillance videos in which it tries to cover almost 13 types of real-world anomalies including arrest, assault, abuse, arson, shooting, fighting, burglary, accident, explosion, shoplifting, vandalism, and stealing. This dataset consists of 950 surveillance videos capturing different types of enlisted anomalies and 950 normal videos. The avenue dataset was provided and used by C. Lu [14] and consists of 37 videos in which 16 were used for training and 21 were used for testing. The UMN dataset [20] consists of five different videos in which abnormal behavior of people walking and running in the opposite direction was captured. The USD Ped1 dataset [58] consists of a total of 70 surveillance videos, where 34 were used in the training phase and the other 36 videos were used for testing. This dataset also tries to capture the anomalous behavior of pedestrians. The USD Ped2 dataset [58] contains 28 surveillance video clips that were also targeted to be used for studies regarding anomaly detection on busy streets. Normal events in this dataset are walking pedestrians, and abnormal events are bikers, wheelchairs, skaters, and joggers. The subway entrance dataset [57] consists of a long video, with a duration of 1 h 36 min, capturing the movement of pedestrians going into the subway station. This dataset offers five types of anomalous events, i.e., loitering (IT), wrong direction walking (WD), irregular interactions among people (II), no payments (NP), and others. The subway exit dataset [57] also consists of a 43-min-long video capturing people coming out of the subway station. The BOSS dataset [46] is a set of surveillance videos captured by the camera in a train. It consists of 12 video clips. The interesting events for anomaly detection in this dataset are panic situations, harassment, and sick people. All these datasets are heavily in use in research in anomaly detection and are being used as a benchmark to compare the results produced by new techniques with previous techniques.

### 3.4. Vehicle Accident Image Dataset (VAID)

After reviewing the available datasets, it was observed that the traffic accident dataset was not available on the shelf, so a new anomaly dataset was constructed based on traffic accidents. The new dataset consists of traffic accident images and videos. The details for all previous datasets are summarized in Table 2, and the statistics for the new dataset are given in Table 3.

#### 3.4.1. Image/Video Collection

To construct the dataset used in this research, images and videos were collected from different sources over the internet. The 278 images in the dataset were acquired directly from different image repositories shot by high-resolution cameras (HRCs) and available over the internet. The rest of the 1082 images were gathered by converting the accident or car crash videos into images by applying a 3rd party video processing tool named DVD Video Soft Free Studio. The videos from which the accident images were captured were the output of a VTSS available over the internet. Another part of the dataset is the testing videos, which were also collected from different sources over the internet and amount to a total of 30 testing videos. The resulting dataset consists of traffic accident events on roads, highways, and motorways, and the vehicles captured having accidents are cars, trucks, buses, and motorcycles.

**Figure 6:**
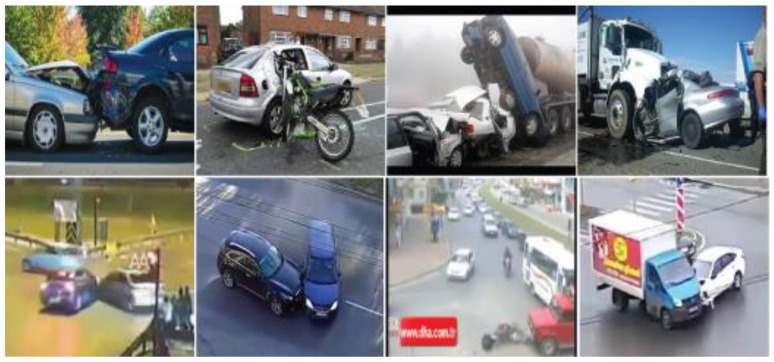
Images of accident dataset. In this dataset, all images consisting of accidents. Some were captured by closed cameras and most were captured by surveillance cameras.

Traffic surveillance cameras capture the accidents in different environments, so to accommodate this effect in our dataset, a part of the collected content was ensured to be in different environmental conditions such as day, night, foggy, and raining. The dataset content also has different resolutions to make the data more diverse. Figure 6 shows a subset of accident images where the first four images were taken directly from available online content and the next four images were extracted from videos collected from a VTSS. 

**Figure 7:**
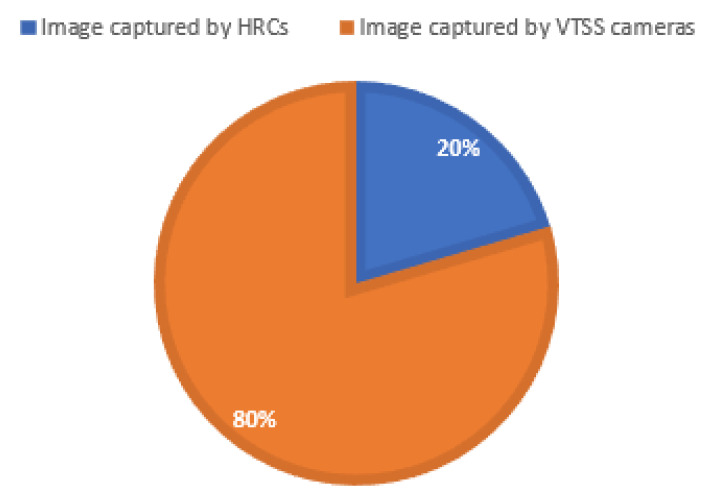
Training data distributions. The VAID dataset was divided into two different categories, training and testing, where 80% of the images were used for the training dataset.

Table 2 describes the overall stats of the newly created dataset, and the distribution is represented in Figure 7.

#### 3.4.2. Pre-Processing of Data

During the pre-processing of the images for training the CNN model, the images were transformed in a series of steps to resolve compatibility issues and to increase the model generalization. The first step in the pre-processing of an input image is swapping the color channels, i.e., RGB channel rearranging to make the image compatible with Keras. The next step is the resizing of the input image to a fixed size, i.e., 224 × 224, without taking the aspect ratio of the image into consideration. In the last step in the pre-processing of an image, mean subtraction is applied to the image, where the mean is calculated for all pixel values in the image and the mean is subtracted from the pixel values. The reason behind this step is to make a standardization to which the other parameters such as weights and biases can refer to. The pre-processing of an image makes the image ready to be provided to a CNN model. In the testing phase of this research, the same set of techniques was applied. The testing of the CNN model was performed on videos, so while testing, the frames of the videos were looped, and all the frames were subjected to the same pre-processing as the training images.

Another technique used in the pre-processing phase of training is data augmentation. This technique is very popular while working with images, as it helps create more data, which helps the computation models to be able to generalize better. In this research, multiple data augmentation techniques were used, i.e., shift, rotation, shear, Flip, etc. Data augmentation was not applicable in the testing phase, as there was no need to multiply data and generate more.

### 3.5. Evaluation Matrices

For analyzing the capability of the implemented method, the following matrices were implemented and used to evaluate our model.

#### 3.5.1. Confusion Matrix

This matrix is used to calculate the degree of error and correctness in classified objects. In the confusion matrix, the TP and TN are a total number of samples that are truly classified, where TP stands for true positive, and TN is true negative. FP and FN are the total numbers of samples that are incorrectly classified, where FP is false positive, and FN is false negative.

#### 3.5.2. Accuracy

Accuracy shows the performance of a model in terms of correctly classified samples. Accuracy can be determined by the confusion matrix, as is it is the ratio of the sum of the truly classified samples, both positive and negative, and the total number of samples in the dataset.

(5) $$Accuracy=\frac{TP+TN}{totat}$$

#### 3.5.3. Precision

Precision is mostly used to determine when the number of samples that are falsely classified as positive is very high. Precision is the ratio of the number of samples that are truly classified as positive and the sum of the number of samples truly classified as positive and falsely classified as positive.

(6) $$Precision=\frac{TP}{TP+FP}$$

#### 3.5.4. Recall

Recall is the ratio of the number of positively classified samples that are actually positive and the total number of correctly classified samples. A recall matrix was used to calculate the number of actual positives the model captured from the testing dataset.

(7) $$Recall=\frac{TP}{TP+FN}$$

#### 3.5.5. F1 Score

The F1 Score is the weighted average of precision and recall. When seeking a balance between the recall and precision an F1 score is needed.

(8) $$F1Score=2\times \left(\frac{\mathrm{Precision}\times \mathrm{Recall}}{\mathrm{Precision}+\mathrm{Recall}}\right)$$

#### 3.5.6. Error Rate

The error rate is the measure of the percentage of total samples that are wrongly classified, either as positive or as negative. This matrix was used to calculate the error rate of the CNN model on the given dataset.

(9) $$Error\;Rate=\frac{FP+FN}{total}$$

### 3.6. Data Flow of the Proposed ML Approach

The data flow (DF) explains the data and their overall flow through the system. The main objective of DF is to present the flow of information about the origin, storage, and ultimate edge of the data. In this research, as data pass through many filters and computation models, showing this flow in a diagram helps in understanding the end-to-end setup. First, images were labeled with accidents, and these images were inserted in the input layer for feature extraction. A model was created and trained on these features of the accident images. After training, the video was provided as a testing input. Finally, the model predicted the video from its features.

## 4. Implementation

### 4.1. Environmental Setup

The environment for the implementation of the methodology is shown in Table 4.

### 4.2. Tool for ML Approach

Python is high-level, versatile, and friendly language. It is highly recommended for the current scientific field of AI, ML, DL, and NLP. Python provides important libraries in deep learning, such as Keras, matplotlib, sklearn, imutils, NumPy, argparse, pickle, cv2, and os, which are helpful in the evaluation and development of deep learning models, general scientific computing, visualizing, and managing the data. For the stated reasons, the python language was used for the implementation of a classifier to find anomalies (accidents) in given input videos.

### 4.3. Data Labeling

An excel file was created to label the data, and the names of all images that existed in the data folder were imported in the excel file. After renaming all images with proper format and numbering, all images were assigned with the label ‘accident’.

### 4.4. Implementation of the DL Approach

For the implementation of the DL approach, a supervised algorithm named convolutional neural networks was used to train the classifier because as it is evident from the literature review and methodology section that the performance of CNNs is high in the classification of images and object detection. The implementation was conducted in a python programming language.

### 4.5. Pre-Processing

After loading an image, pre-processing must be performed on it before using it for training. For Keras and OpenCV compatibility, the pre-processing swapped the color channels and resized the image to 244 × 244 pixels.

### 4.6. Data Augmentation (DA)

For increasing model generalization, data augmentation techniques were used. We initialized two DA objects, one for training and the other for validation. The training object performed flips, zooms, rotation, shears, random rotation, and shifts on the data. Only the mean subtraction technique was performed on the validation data instead of the data augmentation techniques.

### 4.7. CNN

Keras API was used to design the CNN model in two parts. One was the base model, and the other was the head model. For initializing the base model, we used the pre-trained model ResNet50 with imageNet weight because, in our case, we needed to classify input images that had class labels inside the imageNet. At the end of the base model, we specified the input shape of 244 × 244 × 3, which are the input image dimensions for which ResNet50 was originally trained.

*baseModel = ResNet50(weights = “imagenet”, include_top = False,*
*input_tensor = Input (shape = (224, 224, 3)))*

The head model was created on top of the base model, so the output generated from the base model was considered the input of the head model. Moving on, we added a 2D average pooling layer and specified the size of the pool in the x and y directions. The output generated from the previous layer needed to be flattened to enter the fully connected (FC) layer. The FC layer had 512 nodes in our model, which used a rectified liner unit (ReLU) as an activation function. The output layer had the sizes of the classes with Softmax classification. We froze the base model at this stage, so it was not trained via backpropagation.

### 4.8. Compiling and Training of Model

**Figure 8:**
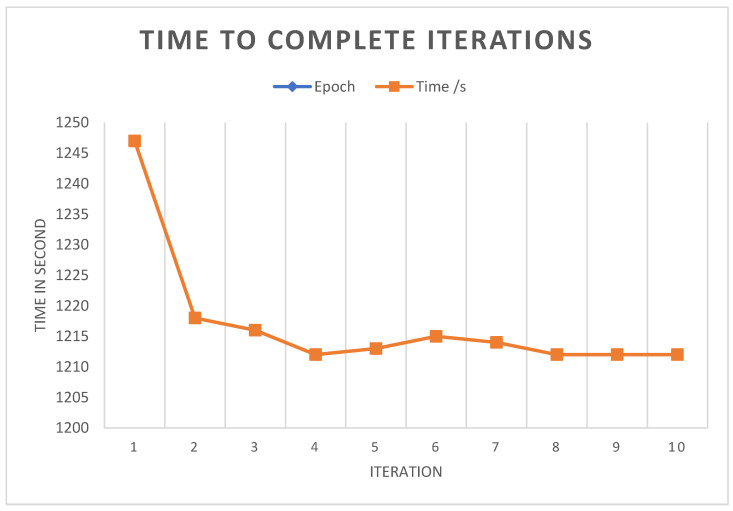
Time, in seconds, to complete the epochs. An epoch is one complete cycle of the model going through the training data.

After developing the architecture of the CNN in Keras, we compiled our model with an optimizer, such as stochastic gradient descent (SGD), and with initial learning and decay learning rates of 1 × 10^−4^. We used “binary cross-entropy” for computing training loss with our classes. In order to train the constructed model on our data, we passed all our training data with trainX and trainY arguments along with 32 batch sizes and 10 numbers of epochs. After the execution of the “fit generator” function in our model, the data were learned by our network with mean substation and data augmentation. After this, we saved our Keras model, which is presented in Figure 8.

#### 4.8.1. Training Result

Here, we trained our model on VAID with 10 epochs using the command given below.

*$ python train.py −−dataset Anomaly − Classifier/data*      −−*model output/activity.model −−label*    −*bin output/lb.pickle −−epochs* 10

#### 4.8.2. Iterations Result

The 10 epochs took a considerable amount of time to complete due to the limitation of resources. The first iteration took 1247 s to complete, and the tenth iteration took 1218 s to execute. The times taken by all iterations are shown in Figure 8.

**Figure 9:**
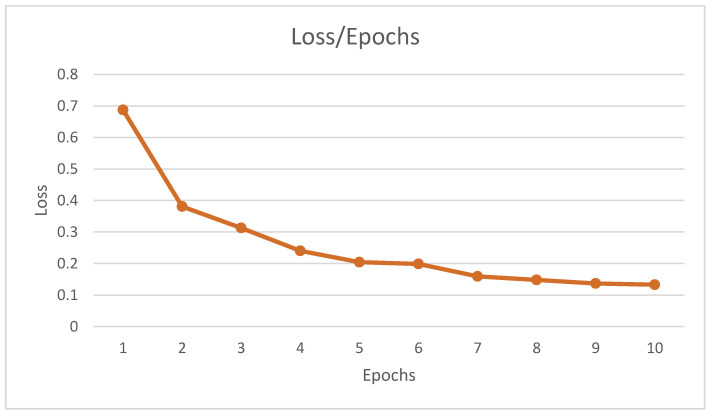
Loss of training model. Training loss is a matrix that is used to evaluate how the model fits the training data. This image represents the error, which was reduced by increasing the epochs.

The training loss of our model was 0.1327. In the first epoch, the training loss was 0.6874, and it decreased with the increasing epochs, which is shown in Figure 9. After the 10th iteration, the training loss was 0.1327.

**Figure 10:**
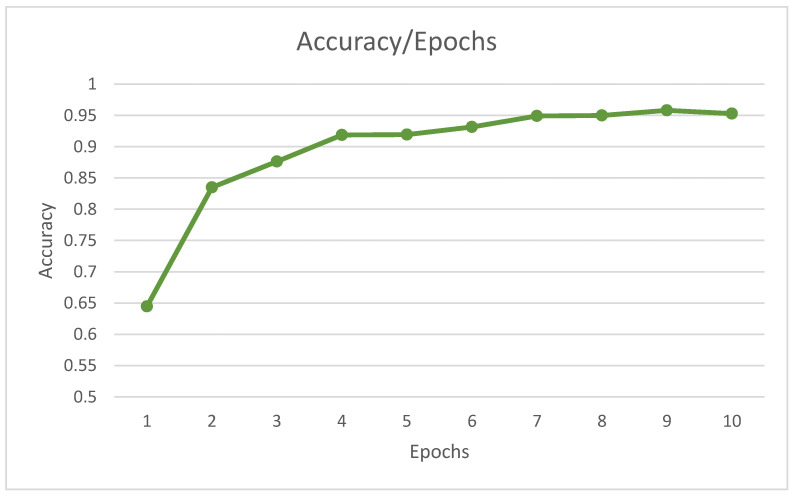
Accuracy of training model. This figure shows the accuracy increased with increasing epochs.

The training accuracy of our model was 0.956, or approximately 95%. Moreover, the accuracy increased as the iterations continued, as shown in Figure 10.

**Figure 11:**
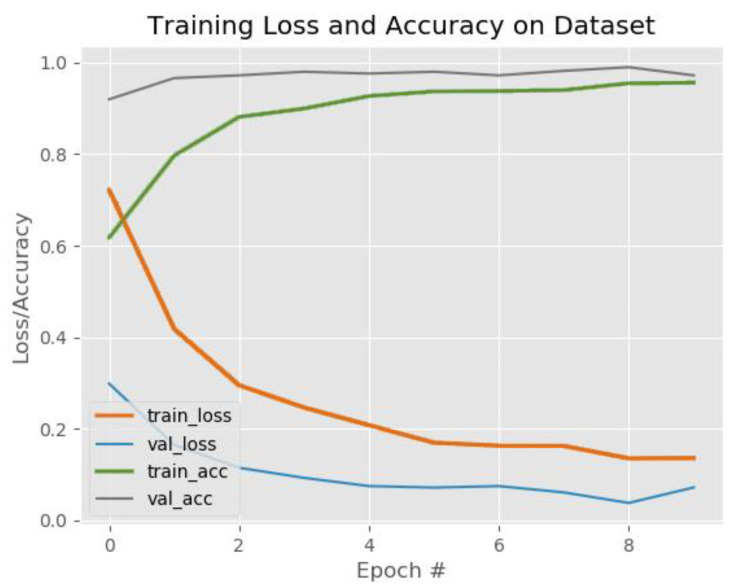
The training loss and accuracy on the VAID dataset. In this figure, the red line represents the training loss, the blue line represents the testing loss, the purple line represents the training accuracy, and the black line represents the testing accuracy.

The overall training loss, training accuracy, validation loss, and validation accuracy on the VAID dataset are shown in Figure 11.

### 4.9. Testing of Model

A previous section in this chapter discusses the process of constructing a CNN model and then training the model on VAID. This part now explains the steps involved in testing the Keras model via a rolling prediction average on the testing dataset. The testing dataset is a collection of accident videos collected from different sources.

For the rolling average algorithm, we loaded the necessary python module and packages such as “deque” and “collections”. Then, we initialized our “cv2.VideoCapture” object and began looping on video frames. We used the VideoCapture class from OpenCV to read frames from our video stream while a loop was used to acquire frames from a video by reading the frames one by one until the end of the video.

#### 4.9.1. Pre-Processing of Frame

In pre-processing a frame, the same steps were performed as during training, including swapping color channels, resizing to fixed pixels, and mean subtraction.

#### 4.9.2. Frame Classification Interface and Rolling Prediction Averaging

Initially, a prediction was made on every frame in the video, but on observing the result, it was seen that the label was flickering rapidly, as not every frame in the video with the occurrence of the anomaly tested positive for the anomaly.

To solve the flickering problem, a prediction was made on every frame in the video, and the result of the prediction was added to an array. The deque function was used to perform a prediction averaging algorithm with a queue size of 128 over the array, resulting in the label class for the rolling average. After that, the NumPy library function was used to find the label with the largest corresponding probability across the average predictions.

#### 4.9.3. Output/Result Label

When the trained Keras model detected an anomaly (accident), it was given testing videos. Then, it drew a predicted label on the frame with the help of a video “writer” using a python library (OpenCV).

## 5. Experiment and Evaluation

### 5.1. Testing Dataset

In the testing dataset, we had 30 videos of accident base and normal flow of traffic, as mentioned in Table 5. All these videos were recorded by cameras used in VTSSs in different places and under different environmental conditions.

**Figure 12:**
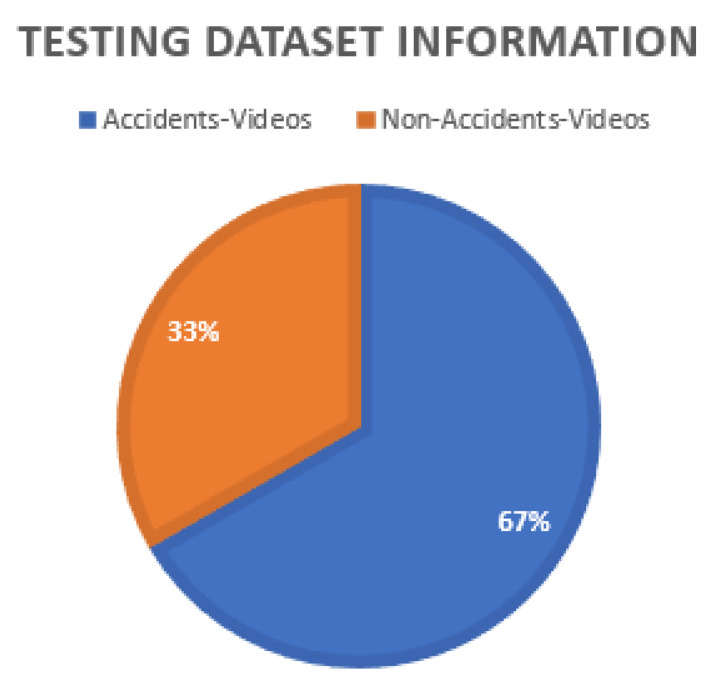
Testing data distribution. The testing data consisted of accident and non-accident videos. The percentage of non-accident videos was 33%, and accident videos were 37%.

The durations of the videos in the testing dataset range from 4 s to 10 s. This dataset is divided into two categories, accidents and non-accidents. The accidents category has 20 videos, and the non-accident category has 10 videos. The distribution of the testing dataset is shown in Figure 12.

**Figure 13:**
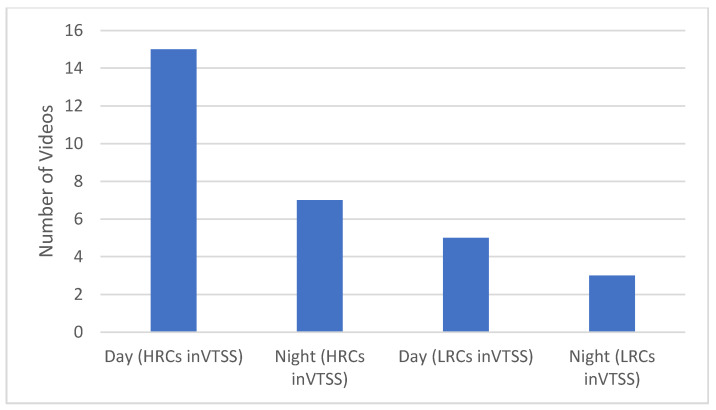
Training videos distributed according to day and night. In this graph, the two different categories are used in two different scenarios: high-resolution cameras and low-resolution cameras in day and night scenarios.

A total of 15 videos in the testing dataset were captured by high-resolution cameras (HRCs) during the day and 7 were captured at night through a VTSS. Moreover, the number of videos that were captured by low-resolution cameras (LRCs) during the day is five and during the night is three. This distribution is shown in Figure 13.

### 5.2. Experimental Results

For evaluating the trained model over unseen data, i.e., for the test dataset we fed the whole testing dataset, which is based on 30 videos, to our trained model one by one and evaluated the results in order to measure the model performance. The model was implemented in a way that replays the input video on a screen and labels the frames of the input video with the detected label. Following are some snapshots of the system capturing scenarios with different situations.

#### 5.2.1. Scenario 1

In the first scenario, we provided a video with the name 001261.mp4, which captured a bus colliding with a car during the day. The model could detect the frame as an anomaly, and it labeled the frame as an activity ‘accident’.

*$ python predict_video.py --model model/activity.model --label-bin model/lb.pickle \* *--input example_clips/001261.mp4 --output output/001261_1frame.avi --size 1*

##### Result

**Figure 14:**
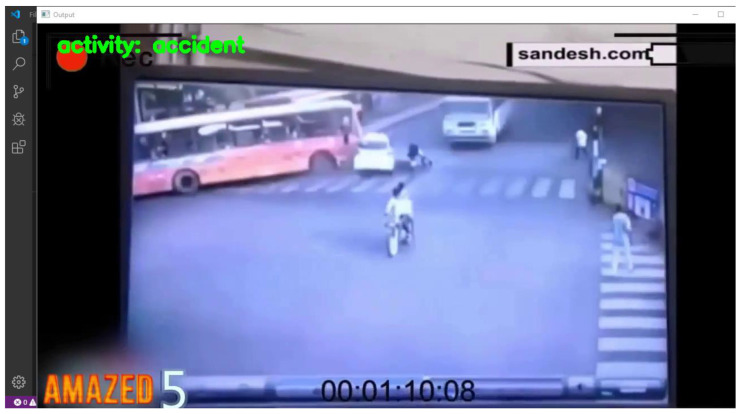
Result captured by test 1. In this scenario, the resolution of the camera was too low, but the viewing angle was in the right direction.

The model correctly labeled it an “accident”. The reason for this could be the clear view of the accident. Otherwise, the quality of the video is not too much better. Moreover, the camera of the VTSS is not installed too far away, as shown in Figure 14.

#### 5.2.2. Scenario 2

In the second scenario, we provided a video with the name acci21.mp4, which captured a car colliding with a car at night. The model could detect the frame as an anomaly, and it labeled the frame as an activity ‘accident’.

*$ python predict_video.py --model model/activity.model --label-bin model/lb.pickle \* *--input example_clips/acci21.mp4 --output output/acci21_1frame.avi --size 128*

##### Result

**Figure 15:**
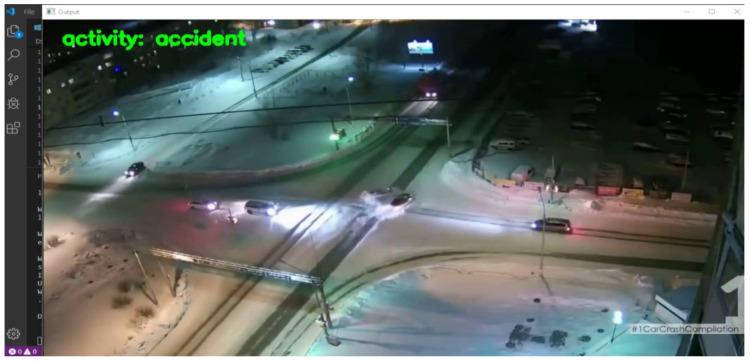
Result captured by test 2. It was correctly labeled as an accident.

In this scenario, the VTSS camera was installed far away from the location where the accident occurred, but the model correctly labeled it an “accident”. Because a VTSS high-resolution camera was installed here, it captured the accident clearly, as shown in Figure 15.

#### 5.2.3. Scenario 3

In the third scenario, we provided a video with the name ambil.mp4, which captured two cars colliding with each other after rain during the day. The model could detect the frame as an anomaly, and it labeled the frame as an activity ‘accident’.

*$ python predict_video.py --model model/activity.model --label-bin model/lb.pickle \* *--input example_clips/ambil.mp4 --output output/ambil_1frame.avi --size 128*

##### Result

**Figure 16:**
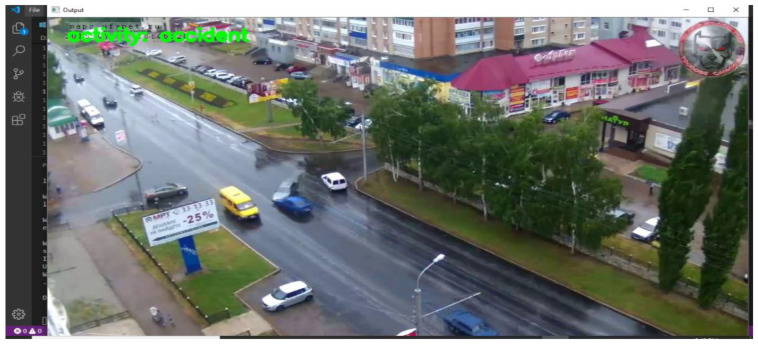
Result captured by test 3 was correctly labeled “accidental”.

In this scenario, the VTSS camera was installed far away from the location where the accident occurred, but the model correctly labeled it an “accident”. Because a VTSS high-resolution camera was installed here, it captured the accident clearly, as shown in Figure 16.

#### 5.2.4. Scenario 4

In the fourth scenario, we provided a video with the name lrpvd.mp4, which captured a jeep colliding with a car. The model could detect the frame as an anomaly, and it labeled the frame as an activity ‘accident’.

##### Result

**Figure 17:**
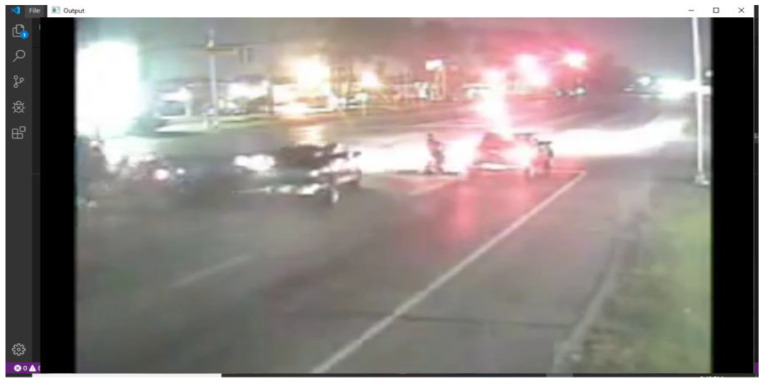
Result captured by test 4. It showed the result without labeling the image.

The model was unable to flag this frame as an accident, but there is an accident captured in this frame. The reason for this could be the poor quality of the image or the abundance of light sources obstructing the view of the accident. It can also be observed that in this frame the detection of an accident is also difficult for a person, as shown in Figure 17.

#### 5.2.5. Scenario 5

In the fifth scenario, we provided a video with the name norm6.mp4, which captured the normal flow of traffic during the day. The model was able to detect that the frame was normal.

*$ python predict_video.py --model model/activity.model --label-bin model/lb.pickle \* *--input example_clips/norm6.mp4 --output output/norm6_1frame.avi --size 128*

##### Result

**Figure 18:**
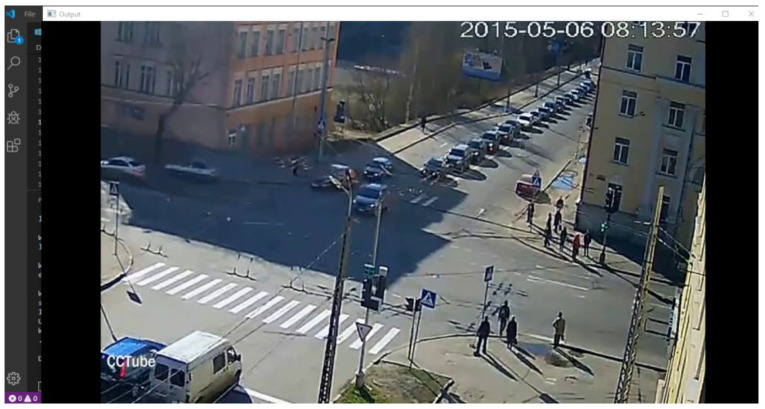
Result captured by test 4. In this image, the model did not detect any accident.

In this scenario, a high-resolution camera of a VTSS was installed in this location. The model correctly labeled it normal, and it is shown in Figure 18.

### 5.3. Results and Discussion

The training dataset consisted of 30 videos of accidents and no accidents (usual traffic). A series of 30 tests was conducted for the validation of the trained model. Every test had a single video for predicting its label. The confusion matrix of these experiments is shown in Table 6.

**Figure 19:**
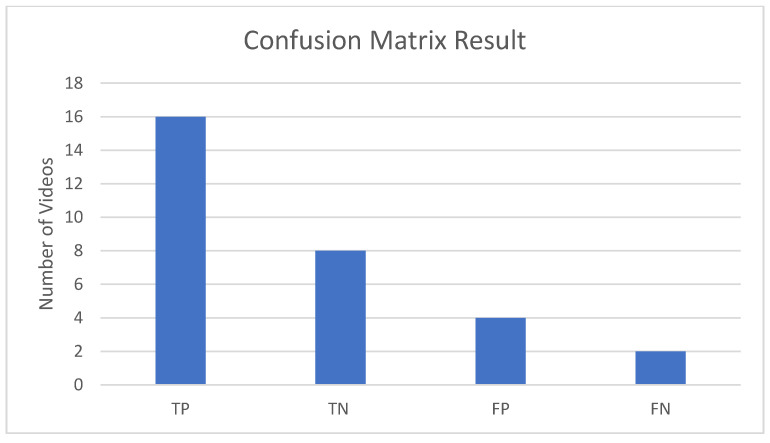
Result of confusion matrix on testing data of vehicle accident image dataset (VAID).

Here, the TP is the number of actual accident videos that were labeled as accidents, and the TN is the number of actual no accident videos that were correctly flagged as normal videos. FP is the number of actual accidents videos where the trained model was unable to detect the accident, and FN is the number of actual no-accident videos that are wrongly classified as accidents. The result of the confusion matrix is shown in Figure 19.

**Figure 20:**
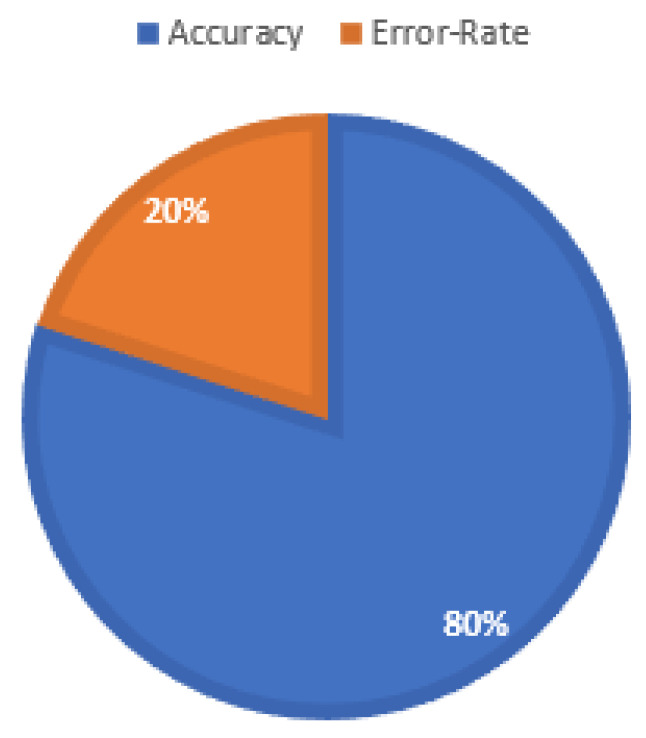
Accuracy measurement of vehicle accident image dataset (VAID). It achieved 80% accuracy on the testing dataset.

The CNN trained model was able to correctly classify 24 videos from the given testing dataset and was unable to correctly classify 6 videos. It was visually observed that the model was unable to correctly classify the videos with low resolutions, with anomalies long distances from the surveillance cameras, foggy environments, and surveillance cameras facing the reflection of light. The accuracy achieved after evaluating the model turned out to be almost 80% for the given dataset, as shown in Figure 20.

**Figure 21:**
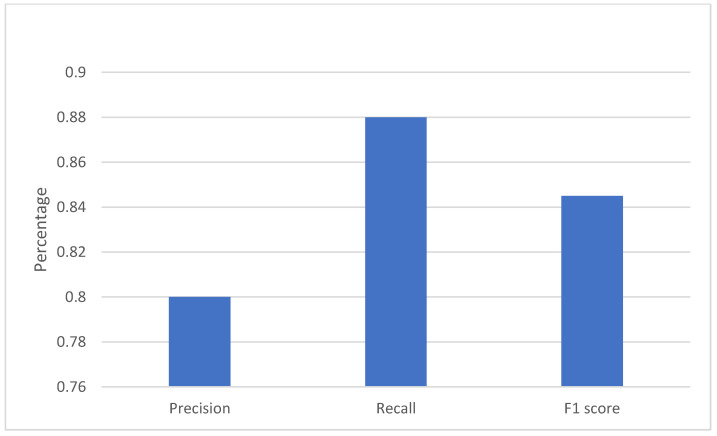
This image presents the results of the precision, recall, and F1 score of the CNN model with rolling prediction algorithms. The precision was 0.8, the recall was 0.88, and the F1 score was 0.85.

Figure 21 shows the precision of 0.8, recall of 0.88, and f1 score of almost 0.845 on the testing data, which implies a good performance of the trained model.

### 5.4. Effect of Rolling Average Prediction Algorithm with CNN and Simple CNN

The VAID dataset was used for both models to obtain results using the evaluation matrix, accuracy, F1 score, recall, and precision. The overall results are shown in Table 7.

### 5.5. The Computational Complexity of Different Approaches

This section examines the computational complexity of our proposed model with simple CNN. Table 8 displays the running times of our proposed model as well as another baseline CNN on the VAID dataset used in training and prediction.

### 5.6. Comparison with Previous Research

Different research was conducted by different researchers to classify the videos using machine learning approaches. In previous research, the naïve Bayes and simple CNN algorithm was used for video classification. This algorithm treats each individual frame of the video as independent from the other frames of the video, hence the implementation of the algorithm faced the “label flickering” problem.

In our proposed approach, the CNN with a rolling prediction averaging was used. This algorithm enables the system to record the last n number of observations, average them, and select the label with the largest probability to find the label for the current frame of video. As discussed in the implementation, the flickering problem was addressed using this algorithm. The assumption when applying this technique to video is that the current frame has similar content to the last n number of observations and not much can change within the time of one frame. As videos are temporal in nature, i.e., the content of video, in most cases, depends on the passing of time, and the videos in the testing dataset were temporal in behavior, this assumption can stand in this research. Hence, the rolling average algorithm is applicable. The use of this algorithm smooths out predictions and makes the proposed solution a better video classifier.

## 6. Conclusions

Deep learning (DL) is a new arena of machine learning (ML) that generally provides significantly accurate results. Deep learning is mostly used to solve complicated problems with the support of deep and complex neural networks. Deep learning techniques have the ability to solve problems that would be very hard or even impossible to solve with previous statistical and machine learning methods. Deep learning proved useful in many fields of science, and currently its techniques are used to solve many practical problems that were difficult to solve before. Deep learning can also be used to find anomalies in different types of data. Its techniques, CNN, RNN, and LSTM, are used to detect or find anomalous data from different datasets. An approach of deep learning CNN has a good reputation for the reorganization of object and image classification in previous research. In this research, CNN was used to find anomalies (accidents) in videos captured by videos of traffic surveillance systems (VTSS). For neural networks, a large set of labeled data is required to perform good training. For that purpose, we created a dataset named the vehicle accident image dataset (VAID) that consists of 1360 images of vehicle accidents. As this dataset was felt to be insufficient for training the model, we used data augmentation techniques such as rotation, shear, zoom, and flip in the training part of the algorithm. The CNN model was constructed by conducting the training on VAID, and to validate and evaluate the model, a dataset consisting of 30 videos was used. During testing, we faced the problem of label flicking between images, and it had a large impact on accuracy. Then, a rolling prediction average algorithm was used along with the CNN trained model to solve the label flickering problem and make it effective for the classification of videos. This research aims to help people who are injured in accidents and the staff that monitor multiple screens of traffic surveillance systems at one time. This system can find an anomaly (accident) through videos and help in notifying the staff to create actions. The proposed research shows good results while testing on videos that were captured by high-resolution cameras used in VTSSs. However, the model was unable to provide efficient results on videos that were captured at large distances and in foggy environments.

A plausible direction for future work can be directed towards finding enhanced models that can cater to the long-distance, poor coverage, and foggy environment videos.

## Figures and Tables

**Table 1:** Summary of different machine learning and deep learning techniques to detect the accidents in different datasets.

Author	Domain	Dataset	Algorithms and Tools
G. Liang [28]	Traffic accident detection using SVM and IoT	Traffic data from the real world such as magnetic field signal and sound signal	An algorithm of ant colony on SVM
V. Ravindean [25]	Detecting road accidents using ML techniques	Captured images from 2 m to 20 m	SVM trained with histogram of matrix features of gradient and grey level co-occurrence
Shivangi-Sharma [26]	Car accident detection using IOT	Real-world data	Arduino IDE, GPS, and GSM with heart rate sensors
N. Kumar et al. [27]	Vehicle accident detection using sensor fusion	1167 observations of variation in speed by conducting the turnover experiment	Naive Bayes (NB), Gaussian mixture model (GMM), and decision tree (DT) techniques
Ren [39]	A deep learning approach to citywide traffic accident risk prediction	Real-world data	Deep learning model of LSTM with improvements
Bortnikov et al. [40]	Accident recognition via 3D CNNs for automated traffic monitoring in smart cities	Real-time traffic videos	A deep learning model 3D convolutional neural network
Tian et al. [41]	An automatic car accident detection method based on cooperative vehicle infrastructure systems	CAD-CVIS dataset	Deep neural network model YOLO-CA
Ohgushi et al. [42]	Road obstacle detection method based on an autoencoder with semantic segmentation	Highway anomaly dataset	Autoencoder with semantic segmentation
Yao et al. [43]	Unsupervised traffic accident detection in first-person videos	Dashboard-mounted camera videos	Future object localization method

**Table 2:** Comparison between datasets on different parameters such as the number of videos, training videos, testing videos, average frames, dataset length, and anomaly type.

Datasets	Numbers of Videos	Training Videos	Testing Videos	Average Frames	Dataset Length	Anomaly Type
UCSD Ped1 [49]	70	34	36	201	5 min	Carts, Bikers, Walking
UCSD Ped2 [49]	28	16	12	163	5 min	Carts, Bikers, Walking
Subway Entrance [48]	1	20 min	-	121,749	1.5 h	NP, WD, IT, II
Subway Exit [48]	1	5 min	-	64,901	1.5 h	NP, WD, IT, II
Avenue [12]	37	16	21	839	30 min	Run, New Object, Throw
UMN [5]	5	3	2	1290	5 min	Run
BOSS [47]	12	8	4	4052	27 min	Panic, Disease, Harassment
UCF [50]	1900	1610	290	7247	128 h	Abuse, Arrest, Fighting, Arson

**Table 3:** Vehicle Accident Image Dataset (VAID). This database was made by collecting accident frames from accident videos. These were collected from different platforms.

Dataset	Training Images	Testing Videos	Anomaly Type
VAID	1360	30	Traffic Accidents

**Table 4:** Setup for experiment.

CPU	Intel Core i5-3570 CPU3.40GHz
Numbers of Cores in CPU	4
Size of Memory	8 GB
Operating System	Window 10

**Table 5:** Testing dataset.

Dataset	Number of Videos	Behavior
Testing	20	Accidents
Testing	10	Non-Accidents

**Table 6:** Confusion matrix of the CNN model with a rolling prediction algorithm. The number of true positives is 16, the number of true negatives is 8, the number of false positives is 4, and the number of false negatives is 2.

N = 30	Predicted Class		
**Actual Class**	**Yes**	**No**	
**Yes**	TP = 16	FN = 2	18
**No**	FP = 4	TN = 8	12
	20	10	

**Table 7:** Result comparison of normal CNN and CNN with rolling average prediction algorithm.

Dataset	CNN	CNN with Rolling Average Prediction Algorithm
Precision	73	80
Recall	79	88
Accuracy	72	82
F1 score	75	85

**Table 8:** Computational complexity of different approaches. The computational complexity of simple CNN was 75% during training and 70% during testing. The computational complexity of CNN with rolling average prediction algorithm was 69% during training and 67% during testing.

Model	VAID DATASET	
	Training	Testing
CNN	75%	70%
CNN with rolling average prediction algorithm	69%	67%

## Data Availability

Not applicable.
